# Antisynthetase syndrome presenting as interstitial lung disease: a case report

**DOI:** 10.1186/s13256-019-2146-0

**Published:** 2019-08-04

**Authors:** Aliena Badshah, Iqbal Haider, Shayan Pervez, Mohammad Humayun

**Affiliations:** Department of Medicine, Khyber Teaching Hospital, Peshawar, Pakistan

**Keywords:** Arthralgia, Myopathy, Interstitial lung disease, Anti-Jo-1 antibodies, Polymyositis, Antisynthetase syndrome

## Abstract

**Background:**

Antisynthetase syndrome is a relatively uncommon entity, and can be easily missed if not specifically looked for in adults whose initial presentation is with interstitial lung disease. Its presentation with interstitial lung disease alters its prognosis.

**Case presentation:**

This case report describes a 27-year-old Pakistani, Asian man, a medical student, with no previous comorbidities or significant family history who presented with a 3 months’ history of low grade fever and lower respiratory tract infections, associated with exertional dyspnea, arthralgias, and gradual weight loss. During these 3 months, he had received multiple orally administered antibiotics for suspected community-acquired pneumonia. When he presented to us, he was pale and febrile. A chest examination was significant for bi-basal end-inspiratory crackles. Preliminary investigations revealed raised erythrocyte sedimentation rate. High resolution computed tomography of his chest showed fine ground-glass attenuation in posterior basal segments of both lower lobes suggestive of interstitial lung disease. He was started on dexamethasone, to which he responded and showed improvement. However, during the course of events, he developed progressive proximal muscle weakness. Further investigations revealed raised creatinine phosphokinase and lactate dehydrogenase. A thorough autoimmune profile was carried out which showed positive anti-Jo-1 antibodies in high titers. A muscle biopsy was consistent with inflammatory myopathy. Clinical, radiological, serological, and histopathological markers aided in making the definitive diagnosis of antisynthetase syndrome. Antisynthetase syndrome is a variant of polymyositis but with visceral involvement, that is, interstitial lung disease and positive anti-Jo-1 antibodies. Our patient responded very well to glucocorticoids and azathioprine.

**Conclusion:**

Antisynthetase syndrome is a rare clinical entity which apart from clinical presentation requires specific serological investigations for diagnosis. Concomitant association of interstitial lung disease gives it a guarded prognosis.

## Introduction

Antisynthetase syndrome (ASS) is a rare, chronic autoimmune disease of undetermined cause. The syndrome is considered a sub-group of idiopathic inflammatory myopathies (IIMs) [1]. IIMs are characterized by different degrees of skeletal muscle inflammation. They are further divided into three sub-groups: (1) sporadic inclusion-body myositis; (2) polymyositis (PM); and (3) dermatomyositis (DM) [2]. ASS is a subset of PM/DM. The hallmark of the disorder is the presence of autoantibodies against the aminoacyl-transfer ribonucleic acid (tRNA) synthetase, also known as antisynthetase antibodies, or anti-ARS [3]. Patients with this syndrome have a characteristic clinical picture consisting of fever, exanthema on the hands (also referred to as mechanic’s hands), myositis, and/or interstitial lung disease (ILD), and/or articular involvement. Raynaud’s phenomenon is frequently observed. The severity and type of pulmonary involvement determines the prognosis of the disease [4, 5].

Anti-Jo-1 was the first anti-ARS discovered. Many other anti-ARS antibodies were discovered later but nearly all have been discovered recently, and only a few laboratories have the facilities to test for them. Since anti-Jo-1 antibodies are readily tested for in patients suspected of ASS, most of the data on ASS are related to presence of anti-Jo-1 antibodies [6].

The strongest predictor of ILD in patients with ASS is the presence of anti-Jo-1 antibodies. Nearly 70% of patients with ASS with ILD have detectable anti-Jo-1 antibodies. In patients with ASS with ILD, disease activity is strongly related to the titers of anti-Jo-1 antibodies. Other less common variants of anti-ARS antibodies include: anti-PL-7, anti-PL-12, anti-OJ, anti-EJ, anti-KS, anti-ZO, and anti-tyrosyl antibodies [6].

We discuss the case of a patient who initially presented with features of ILD, but subsequently developed musculoskeletal features that could not be explained on the basis of ILD alone. Further investigations led to the diagnosis of ASS in the patient. We hope that this rare presentation will add to medical literature and aid in early diagnosis of other patients presenting with similar features.

Autoimmune conditions have a high prevalence in the Western world when compared to Eastern countries. ASS, though a well-known and established entity, is rarely seen in the South Asian part of the world, and so the purpose of this case report is to highlight the importance of keeping in mind this and other autoimmune conditions in our patients when common conditions with similar presentations have been excluded.

## Case presentation

**Fig. 1:**
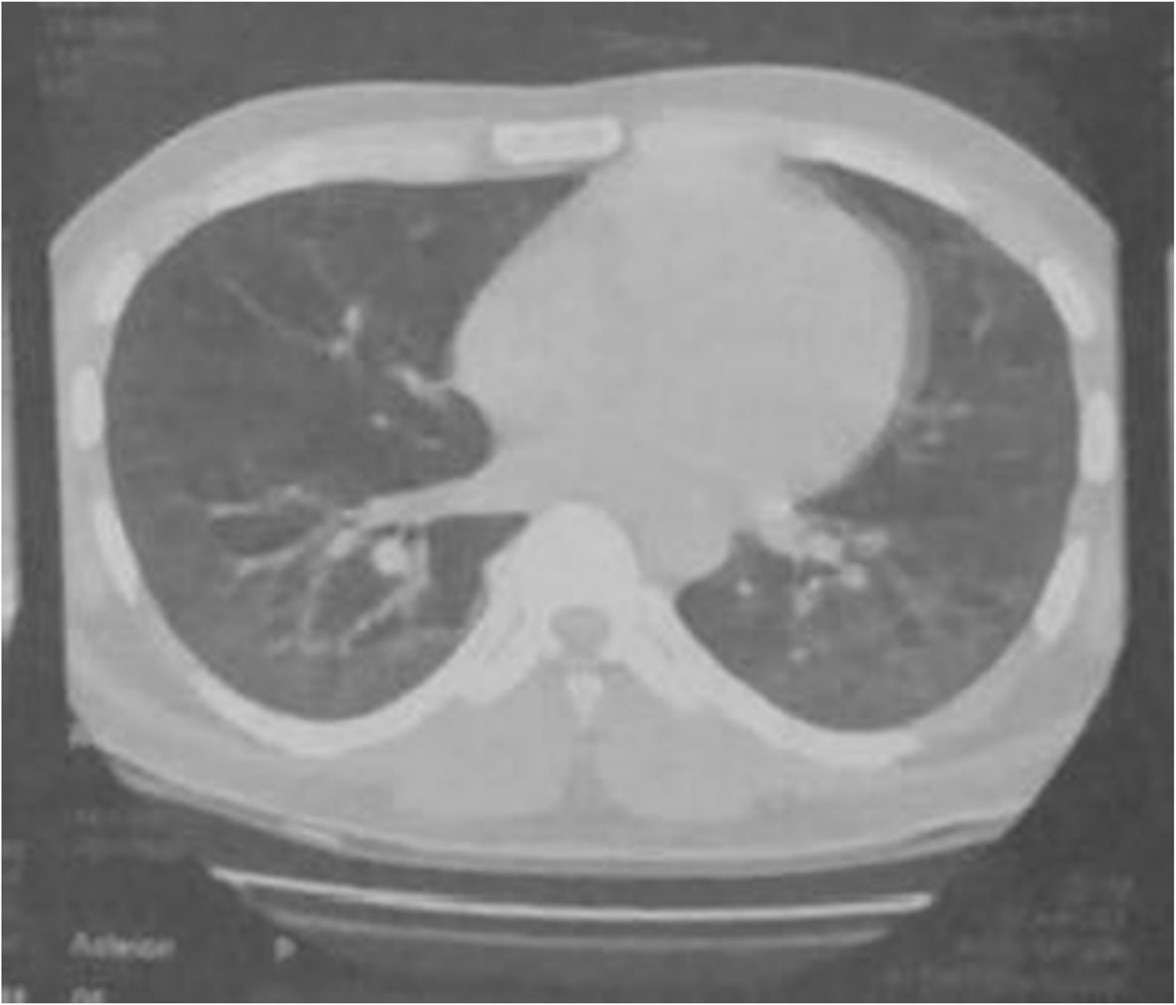
CT scan showing features suggestive of Interstitial Lumg Disease (ILD)

**Fig. 2:**
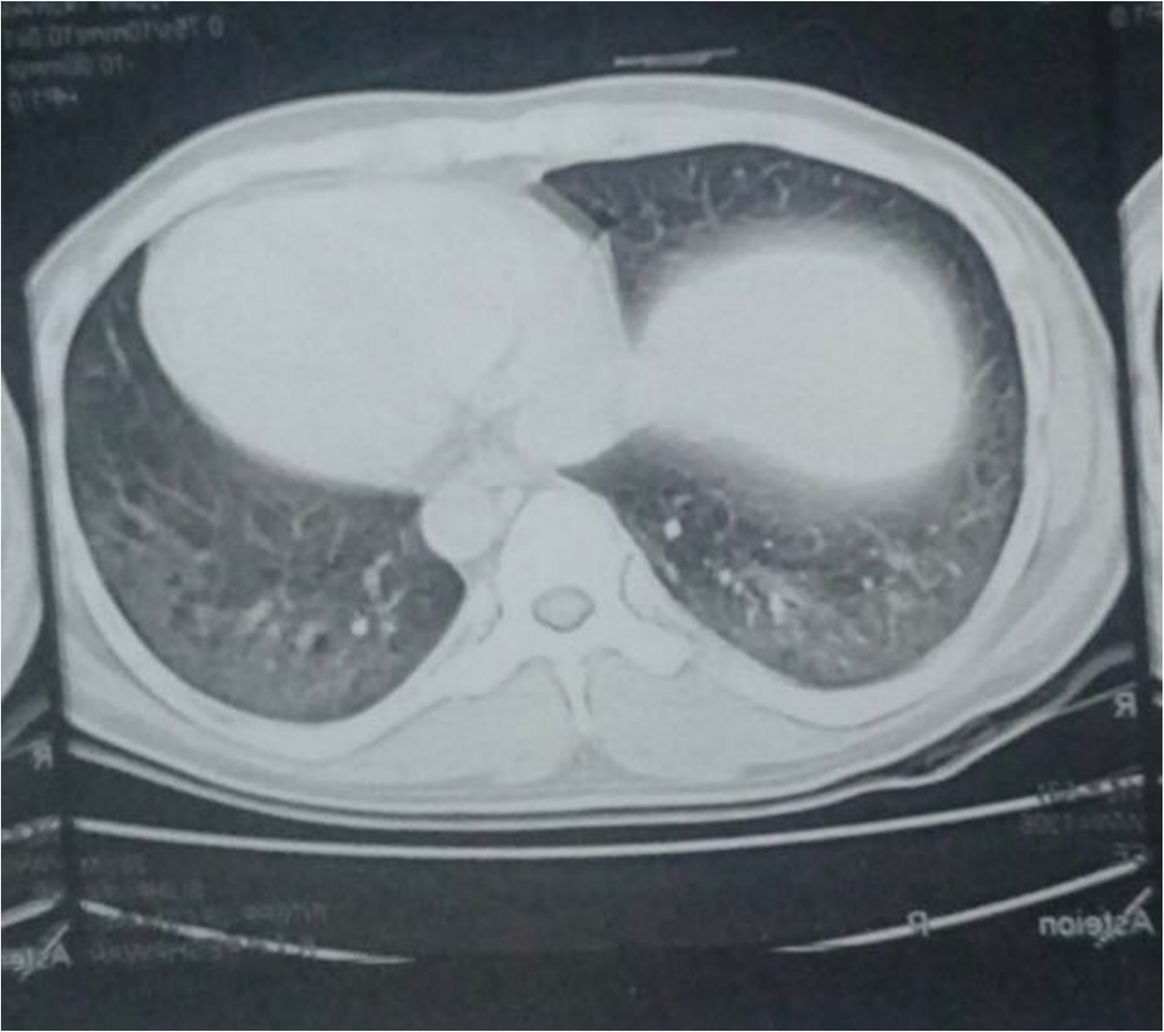
CT scan showing areas of ground glass haze and traction bronchiectasis

A 27-year-old Pakistani, Asian man, a medical student, with no previous comorbidities and no past history of tobacco smoking and alcohol intake, presented with 3 months’ history of frequent bouts of lower respiratory tract infections associated with exertional dyspnea, arthralgias, gradual weight loss, low grade fever, easy fatigability, and anorexia. His family history was also insignificant for any respiratory or other systemic pathology. In the fourth month of his illness, his fever became high grade associated with profuse sweating. All the baseline investigations were carried out on an out-patient basis. His white cell count was raised and a chest X-ray showed basal consolidation. A suspicion of pneumonia was made and he was started on broad-spectrum antibiotics. His condition improved temporarily but the symptoms re-emerged after a few days. He also received a course of orally administered fluoroquinolones but there was no improvement. A subsequent high resolution computed tomography (HRCT) scan of his chest revealed bilateral ground-glass haziness with areas of traction bronchiectasis, more so in the posterior basal segments of both lower lobes, suggestive of ILD (Figs. 1 and 2). He was given dexamethasone which improved his respiratory symptoms. In the sixth month of illness, he developed progressive proximal muscle weakness. He had difficulty in rising up from a chair and had difficulty in rising up from a squatting position. Throughout this period he had progressive weight loss (19 kg lost in 2 months) and profuse sweating. He was admitted to our hospital and thoroughly investigated. With time his proximal muscle weakness became severe and defined. It now involved his upper limbs as well and he had difficulty in combing his hair. He also gave a history of painful and cyanosed finger tips in winter.

He had lost muscle bulk (19 kg lost in 2 months) and was pale. He was febrile with recorded fever of 39.4 to 40.0 °C (103–104 °F). There was symmetric arthralgia but no arthritis. A chest examination still revealed bi-basal inspiratory crackles.

Routine and specialized investigations were carried out, which are tabulated in Table 1.

**Table 1:** Routine and specialized investigations of the patient

Investigations	Results	Normal range
Serial white cell counts	14,900; 15,900; 24,900; 28,100/cmm	4000–11,000/cmm
C-reactive protein (CRP)	24 mg/l	< 3 mg/l
Creatinine phosphokinase (CPK)	6680 U/l	24–175 U/l
Serial erythrocyte sedimentation rate (ESR)	90; 40; 30 mm/first hour	0–15 mm/first hour
Serial lactate dehydrogenase (LDH)	2030; 614; 1391 U/l	140–250 U/l
Liver/renal profile/serum uric acid	Within normal limits	–
Peripheral smear/reticulocyte count	Neutrophilic leukocytosis/0.5%	–
Malarial parasite	Not detected	–
Serum calcium	Within normal	–
Serum angiotensin-converting enzyme (ACE) levels	Within normal	–
Viral serology (HBV, HCV, HIV)	Negative	–
*Brucella* serology	Negative	–
Venereal Disease Research Laboratory (VDRL) test	Negative	–
RA factor	Negative	–
Anti-neutrophilic antibody (ANA)	Negative	–
Serum anti-double-stranded DNA (anti Ds-DNA), anti-smooth muscle antibodies (ASMA), and anti-mitochondrial antibodies (AMA)	Non-reactive	–
Serum complement levels	Within normal	–
Thyroid function tests	Within normal	–
Blood/urine culture	No growth yielded	–
Echocardiography	Normal	–
Parathyroid hormone (PTH) levels	42 pg/ml	7–53 pg/ml

*CRP* C-reactive protein, CPK creatinine phosphokinase, *ESR* erythrocyte sedimentation rate, *LDH* lactate dehydrogenase, *ACE* angiotensin converting enzyme, *HBV* hepatitis B virus, *HCV* hepatitis C virus, *HIV* human immunodeficiency virus, *VDRL* Venereal Disease Research Laboratory, *RA* rheumatoid arthritis, *ANA* Anti-neutrophilicantibody, anti-dS *DNA* Anti-double stranded DNA, *ASMA* Anti-smooth muscle antibodies, *AMA* anti-mitochondrial antibodies, *PTH* parathyroid hormone

A bone marrow biopsy was done to exclude any myeloproliferative disorder that could have been responsible for the continuously raised total leukocyte count (TLC). It turned out to be normocellular with no evidence of leukemia or lymphoma. His 25-hydroxy vitamin-D was deficient with a value of 4.9 ng/ml (normal value, 30–100 ng/ml; severe deficiency, < 20 ng/ml). An ultrasound of his abdomen was suggestive of only cholelithiasis, with no pleural or pericardial effusions. Bronchoalveolar lavage for acid-fast bacilli (AFBs), GeneXpert, and AFB culture/sensitivity and cytology was negative. A muscle biopsy was consistent with inflammatory myopathy.

Since his musculoskeletal features could not be explained by ILD alone, a full autoimmune profile was sent: extractable nuclear antigen (ENA) profile. Anti-Scl 70 immunoglobulins G (IgG), anti-SRP IgG, anti-Mi2 IgG, anti-PL-7, and anti-PL-12 were negative. Anti-Jo-1 antibodies turned out to be positive in titers of 81 AU/ml (normal range 6–12 AU/ml). The clinical features in conjunction with elevated muscle enzymes like creatinine phosphokinase (CPK), anti-Jo-1 positivity, muscle biopsy, and HRCT findings led to a presumptive diagnosis of ASS. He was commenced on pulsed intravenous methyl prednisolone 1 gm daily for 3 days followed by orally administered prednisolone (1 mg/kg body weight), and vitamin D3 and calcium supplements at our hospital. He responded to the treatment. His CPK reduced and symptoms improved. He was discharged on orally administered glucocorticoids, orally administered azathioprine, proton pump inhibitors, and vitamin D3 analogs (to prevent corticosteroid-induced osteoporosis). Six months later, he is on a maintenance dose of orally administered steroids and azathioprine and is symptom free. The case is being presented after informed consent from the patient.

## Discussion

ASS is a rare chronic autoimmune disease that clinically manifests with inflammatory myopathies, ILD, inflammatory arthritis, skin hyperkeratosis (mechanic’s hands), and Raynaud’s phenomenon [4]. The pathophysiology is not entirely understood. However, the autoantibodies against the aminoacyl-tRNA synthetase appear to be linked to the cause of this syndrome. These autoantibodies may arise after viral infections or patients may have a genetic predisposition [3, 5]. The most common among antisynthetase antibodies is anit-Jo-1 which is produced against the antigen histidyl-tRNA synthetase. The less common antibodies are PL-7, PL-12, OJ, EJ, KS, WA, YRS, and Zo. The prevalence in the general population remains unknown [5]. The reported annual incidence of PM/DM ranges from 2 to 10 new cases per million persons; the prevalence of antisynthetase antibodies is 20 to 40% of total PM/DM population. The incidence of anti-Jo-1 positive cases ranges from 1.2–2.5 per million. The prevalence is nearly 1.5 per 100,000 population [6]. The disease mainly affects adults (average age 50 years with a range of 22–74 years) with females more prone to the syndrome than males by a ratio of 2–3:1 [1, 2]. Myositis occurs in more than 90% of cases. Weakness of the muscles of mastication can result in aspiration pneumonia, while weakness of respiratory muscles can lead to dyspnea or respiratory failure [3].

ILD has been seen in 60% of patients. ILD presents with sudden or gradual onset of exertional dyspnea and difficult-to-control dry cough. It can complicate into pulmonary hypertension. The usual type of ILD seen in ASS is nonspecific interstitial pneumonia (NSIP). Cryptogenic organizing pneumonia (COP) and fibrosing alveolitis are also associated with ILD. Those with COP (previously known as bronchiolitis obliterans with organizing pneumonia – BOOP) have a better prognosis than those with either diffuse alveolar lesions or interstitial pneumonia. ILD is investigated by pulmonary function tests (PFTs) and HRCT scans of lungs [4]. ILD is subdivided into three sub-groups based on HRCT findings: COP characterized by consolidation and linear opacities, NSIP characterized by ground-glass opacities, and usual interstitial pneumonia (UIP) characterized by honeycomb pattern and traction bronchiectasis [6].

Inflammatory arthritis is an associated feature in 50% of the patients; it is characterized by symmetric polyarthritis of small joints of the hands and feet. It is usually non-erosive and non-deforming, but rarely can present as erosive and deforming arthritis. ASS should be considered in the differential diagnosis of sero-negative rheumatoid arthritis, because both these conditions behave similarly [7]. The diagnosis of ASS requires the presence of autoantibodies along with two major criteria or one major and two minor criteria from the following list:

major criteria [8]

- ILD (occurring without a known cause)
- PM or DM

minor criteria

- arthritis
- Raynaud’s phenomenon
- mechanic’s hands.

Muscle enzymes (CPK and aldolase) are raised. CPK levels can reach 5–50 times the upper limit of normal levels. In cases of excessive muscle wasting, CPK may stay within normal range despite active muscle inflammation [7]. This is due to reduced muscle bulk.

Clinical features are more important than laboratory investigations in the diagnosis of ASS. If anti-Jo-1 antibodies are negative, a search for non-Jo-1 antibodies can be made in patients with clinical ASS [2, 5]. Concomitant occurrence of ILD and myositis increases the likelihood of positive anti-Jo-1 antibodies. The PFTs will show a restrictive pattern in ASS in which the forced vital capacity is less than 80% and the diffusing capacity of carbon monoxide is less than 70% of the predicted value. HRCT of the chest shows various patterns of lung injury, the most common being NSIP. Patients with ASS and active myositis should be investigated for ILD, because ILD determines the ultimate prognosis of the disease [6].

Muscle biopsy aids in the diagnosis of ASS but negative biopsy does not exclude ASS if there is strong clinical suspicion. Histopathology reveals predominant perimysial inflammation. Macrophages and lymphocytes dominate the inflammatory cells in contrast to PM and DM where predominant cells are lymphocytes only. Both muscle degeneration and regeneration are observed at the same time [9].

Electromyography differentiates between weakness of myopathic and neuropathic origin. It can also hint at the presence of inflammatory myopathies. However, it is a nonspecific test. Magnetic resonance imaging (MRI) of affected muscles is rarely ordered because it lacks specificity and sensitivity [5, 6].

The literature supports the fact that ASS heralds the presence of a malignancy. Some case reports have mentioned different malignancies cropping up within 6–12 months of the diagnosis of ASS. Age-appropriate screening is therefore recommended for all patients with ASS. Glucocorticoids are the first line of treatment. Prednisone is initially given at high doses (1 mg/kg per day) for 4–6 weeks to achieve disease control; then tapered slowly over 9–12 months to the lowest effective dose to maintain remission and also to avoid the undesired effects of steroids. In more severe cases, pulsed intravenous methyl prednisolone 1000 mg daily for 3–5 days may be necessary [10]. Remission is achieved in 25–68% of cases [8]. Prophylactic treatment in the form of calcium and vitamin D supplements and bisphosphonates is recommended against steroid osteoporosis.

Immunosuppressive therapy is initiated as steroid-sparing therapy or to reduce the dose of steroids effective for immune suppression and symptom improvement [7]. Multiple randomized clinical trials have confirmed better efficacy of combined prednisolone and azathioprine therapy than isolated steroid therapy [8]. Severe pulmonary involvement not responding to steroids and azathioprine qualifies for the use of monthly intravenous infusion of cyclophosphamide.

Intravenous immunoglobulin (IVIG) therapy is recommended in patients who fail to respond to combined steroid and azathioprine therapy. It is also indicated in patients not fit for cytotoxic drug therapy. Thirdly, it is indicated in patients with esophageal involvement manifesting as dysphagia and other motility problems [10]. Patients who fail to respond to steroids, methotrexate/azathioprine, and IVIGs may be considered for rituximab as first-line therapy [11]. Physical therapy and rehabilitation play their role in reducing further muscle wasting from disuse and preventing muscle contractures.

Serum CPK levels can take much longer to normalize, therefore, symptomatic improvement is a better marker of response to treatment than laboratory investigations. If we rely on CPK levels as the sole marker of improvement in symptomatology, this may expose the patients to unnecessary high doses of steroids for prolonged periods, thereby exposing them to the side effect profile of prolonged high-dose corticosteroid therapy. Age greater than 60 years, concomitant presence of malignancy, negative anti-neutrophilic antibody (ANA) antibody, and severity and extension of lung disease are markers of poor prognosis. Delay in diagnosis and treatment also leads to poor prognosis. Patients who are Jo-1-negative have worse survival rates than patients who are Jo-1 positive [12, 13].

## Conclusion

This case report stresses the importance of suspecting ASS in patients presenting with unexplained ILD. The diagnosis has important prognostic and therapeutic implications. It is not always possible to make an early diagnosis because the clinical presentation is varied and very often nonspecific. Myositis-specific antibody tests are not carried out in the initial workup of patients presenting with predominantly ILD features. There is considerable clinical heterogeneity and one or other manifestation can predominate or can be the only expression of the syndrome. Furthermore, in the same patient different features can prevail at different times and may even develop years after the onset of the disease.
